# Teaching case 1-2020: Adult-onset leukoencephalopathy with axonal spheroids and pigmented glia – An unusual cause of dementia 

**DOI:** 10.5414/NP301253

**Published:** 2019-12-03

**Authors:** Sigrid Klotz, Franz Riederer, Nora Hergovich, Thomas Schlager, Lara Steinkellner, Elisabeth Fertl, Cristoph Baumgartner, Alexander Zimprich, Ellen Gelpi

**Affiliations:** 1 Institute of Neurology, Medical University of Vienna,; 2 Neurological Center Rosenhuegel and Karl Landsteiner Institute for Epilepsy Research and Cognitive Neurology,; 3 Department of Neurology, Rudolfstiftung Hospital,; 4 Department of Oncology, Krankenhaus Hietzing,; 5 Department of Neurology, Medical University of Vienna, Vienna, Austria,; 6 Neurological Tissue Bank of the Biobanc-Hospital Clinic-IDIBAPS, Barcelona, Spain, and; 7 University of Zurich, Faculty of Medicine, Deparment of Neurology, Zurich, Switzerland

**Keywords:** leukodystrophy, ALSP, adult onset leukodystrophy, axonal spheroids, pigment

## Abstract

No abstract available.

We present an unusual cause of dementia in a man in his fifties. It started at the age of 51 with personality change, followed by prolonged generalized tonic-clonic seizures, non-convulsive status epilepticus, and gradual cognitive deterioration characterized by generalized slowing, marked acalculia, aphasia, apraxia, and frontal dysfunction, associated with dopamine irresponsive parkinsonism. MRI showed bilateral white matter hyperintensities on T2-weighted images without contrast enhancement and atrophy involving the corpus callous. Although extensive diagnostic efforts were made including genetic testing for CADASIL, lysosomal and peroxisomal disorders, Huntington’s disease, etc., no etiologic diagnosis was achieved. Differential diagnoses included mitochondrial encephalomyopathy, cerebral vasculitis, (autoimmune-) encephalitis, among others. The patient died due to aspiration pneumonia aged 58 years. Gross brain examination revealed a severe bilateral frontally and parietally accentuated white matter softening with yellowish discoloration (Figure 1A). This was associated with cortical and subcortical atrophy and ventricular enlargement with relative sparing of brainstem and cerebellum. Histology showed a severe diffuse supratentorial leukoencephalopathy (Figure 1B) with myelin (Figure 1C) and axonal loss, loss of oligodendrocytes, and reactive gliosis. The reactive glial cells partly exhibited cytoplasmic pigment accumulation without metachromasia (Figure 1D, E, F). In better preserved areas there was an abundance of axonal spheroids (Figure 1G, H, I). Subcortical U-fibres were relatively preserved (Figure 1C), and deep cortical layers showed focally the appearance of a laminar pseudonecrosis. No abnormal neurodegeneration-related protein aggregates were detected. Based on these features, the diagnosis of “adult-onset leukoencephalopathy with axonal spheroids and pigmented glia” (ALSP) was made. 

Adult onset leukodystrophies are a group of clinically and pathophysiologically heterogenous diseases [1, 5]. Typical symptoms are cognitive and movement dysfunction as well as personality change and seizures, like in our case. Due to the heterogenous clinical picture, the rareness of the disorders, and the clinical overlaps with other diseases, the clinical diagnosis remains still challenging. Advances in genetic analyses helped to further characterize the spectrum of leukoencephalopathies [2]. The underlying disease mechanism of the different leukoencephalopathies varies greatly and is still not fully elucidated and understood [1]. The involvement of oligodendrocytes, microglia, astrocytes and neurons, as well as vessels have been described. The spectrum ranges from primary demyelinating processes to primary axonopathies [1, 3]. 

ALSP, manifesting as genetic or sporadic disorder, is the most common non-vascular leukoencephalopathy with an onset in adulthood. It comprises previous distinct disease forms such as hereditary diffuse leukoencephalopathy with spheroids (HDLS) and pigmentary orthochromatic leukodystrophy (POLD) [4]. The typical neuropathological features include abundance of axonal spheroids and glia with pigment accumulation [4, 5]. Typically, the axonal spheroids can be seen in early lesions rather than in areas with extensive damage [6]. Mutations in the *colony stimulating factor receptor (CSF1R)* – gene have been identified in several cases, a gene involved in microglial function. In fact, a scarcity of microglia and morphological alterations have been described in autopsies of patients with ALSP [7], although the exact connection between microglial dysfunction and white matter depletion needs further exploration. Recently, diagnostic criteria for ALSP due to *CSF1R* have been proposed and include five “core features”, three “exclusionary findings”, and four “supportive findings”, comprising age at onset, various clinical signs, neuroimaging, and genetic data [8]. The patient presented here would have fulfilled the criteria for probable ALSP as all five core features apply and no genetic testing for *CSF1R* mutation was done. 

This case highlights the diagnostic challenges this disease poses and the importance of genetic testing and detailed postmortem neuropathological examination in unclear neurological situations. It should also serve as a reminder that rare diseases such as ALSP may be an uncommon cause of dementia in fairly young patients. 

## Funding 

SK is partially funded by the Austrian Ministry of Health “Bundesministerium für Arbeit, Soziales, Gesundheit und Konsumentenschutz“ for CJD surveillance – ÖRPE. There is no specific funding for the project. 

## Conflict of interest 

The authors report no conflict of interest. 

**Figure 1.**
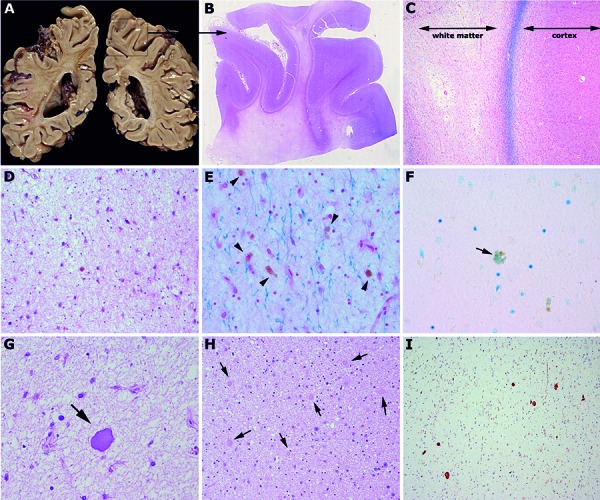
A: Coronal sections through the brain hemispheres show prominent white matter rarefaction with yellowish discoloration. B, C: Klüver-Barrera stain reveals the severe leukoencephalopathy with relative preservation of U-fibres. D: At higher magnification there is prominent rarefaction of white matter with loss of oligodendrocytes and reactive astrocytes. These show partly brownish pigment in the cytoplasm (E, arrows) without metachromasia (F, toluidin blue). G, H, I: Presence of abundant and partly large axonal spheroids (arrows) that are also well identified with antibodies against neurofilaments (I).
