# Teaching case 1-2020 – ADDENDUM: Adult-onset leukoencephalopathy with axonal spheroids and pigmented glia due to a novel *CSF1R* mutation – An unusual cause of dementia 

**DOI:** 10.5414/NP301449

**Published:** 2021-11-30

**Authors:** Sigrid Klotz, Franz Riederer, Nora Hergovich, Thomas Schlager, Lara Steinkellner, Elisabeth Fertl, Christoph Baumgartner, Matias Wagner, Alexander Zimprich, Ellen Gelpi

**Affiliations:** 1 Division of Neuropathology and Neurochemistry, Department of Neurology, Medical University of Vienna, Austria,; 2 Neurological Center Rosenhuegel and Karl Landsteiner Institute for Epilepsy, Research and Cognitive Neurology,; 3 Department of Neurology, Clinic Landstraße,; 4 Department of Oncology, Clinic Hietzing,; 5 Department of Neurology, Medical University of Vienna, Vienna, Austria, and; 6 University of Zurich, Faculty of Medicine, Department of Neurology, Zurich, Switzerland,; 7 Institute for Neurogenomics, Helmholtz Zentrum, Munich, Germany, and; 8 Institute of Human Genetics, Technical University Munich, Munich, Germany

## Abstract

Not available.

Sir, – In the issue “Vol. 39 – No. 1/2020” of *Clinical Neuropathology*, we described the neuropathological features in a male patient in his fifties with an early-onset dementia. We entitled the article “Adult-onset leukoencephalopathy with axonal spheroids and pigmented glia – An unusual cause of dementia [1]”. 

At the time of the publication, genetic testing was not available. Fortunately, we have now been able to analyze an archival blood sample and to perform whole exome sequencing (Institute of Human Genetics, Technical University Munich, Germany). A novel heterozygous missense variant c.2546T>C, p. (Phe849Ile) in the *CSF1R* (colony stimulating factor-1 receptor, NM_005211.3) gene was detected. This variant is considered as “likely pathogenic” according to the American College of Medical Genetics and Genomics (ACMG) criteria [2]. 

Our genetic findings can confirm that the described case was a “definite” case of adult-onset leukoencephalopathy with axonal spheroids and pigmented glia due to *CSF1R* mutation [3]. 

## Funding 

No specific funding for this work. 

## Conflict of interest 

The authors declare no conflict of interest.

**Figure 1:**
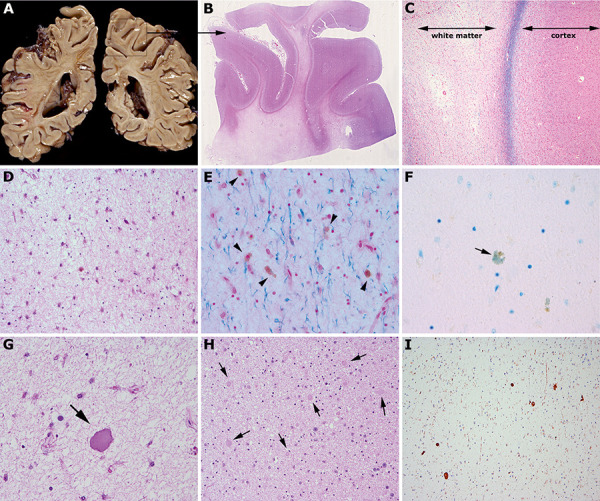
A: Coronal sections through the brain hemispheres show prominent white matter rarefaction with yellowish discoloration. B, C: Klüver-Barrera stain reveals the severe leukoencephalopathy with relative preservation of U-fibers. D: At higher magnification, there is prominent rarefaction of white matter with loss of oligodendrocytes and reactive astrocytes. These show partly brownish pigment in the cytoplasm (E, arrows) without metachromasia (F, toluidine blue). G, H, I: Presence of abundant and partly large axonal spheroids (arrows) that are also well identified with antibodies against neurofilaments (I).
